# Integrated analysis of miRNA and mRNA expression profiles in the bursa of Fabricius of specific pathogen-free chickens infected with avian reticuloendotheliosis virus strain SNV

**DOI:** 10.1016/j.psj.2025.104847

**Published:** 2025-01-23

**Authors:** Yubo Zhao, Qing Zhang, Meng Wang, Bingrong Wu, Saisai Zhao, Xinhui Wei, Youxiang Diao, Yi Tang, Jingdong Hu

**Affiliations:** a College of Veterinary Medicine, Shandong Agricultural University, No.7 Panhe Street, Tai'an 271017, China; b College of Animal Science and Technology, Shandong Agricultural University, No.7 Panhe Street, Tai'an 271017, China; c Institute of Animal Science, Chinese Academy of Agricultural Sciences, China

**Keywords:** reticuloendotheliosis virus strain SNV, miRNA, mRNA, integrated analysis

## Abstract

Reticuloendotheliosis virus (REV) is a gamma retrovirus that can cause immunosuppression, dwarf syndrome and acute reticulocytoma in poultry. The molecular mechanism by which REV infection leads to immunosuppression and tumorigenesis is poorly understood. In this study, we elucidated the regulatory network of miRNA-mRNA and the major signaling pathways involved in REV-SNV infection. Therefore, we used the spleen necrosis virus (SNV) model of REV to inoculate one-day-old specific pathogen-free (SPF) chickens and then performed global miRNA and mRNA expression profiling by conducting high-throughput sequencing of 18 bursa of Fabricius samples collected at 7, 14, and 21 dpi. In total, 213 differentially expressed miRNAs (DEMs) and 3311 differentially expressed genes (DEGs) were identified. In the miRNA-mRNA network constructed based on the association analysis of these DEMs and DEGs, 1376 negatively correlated miRNA-mRNA pairs were identified; among them, 82 pairs were identified at 7 dpi, 203 pairs were identified at 14 dpi, and 873 pairs were identified at 21 dpi. Moreover, the results of the Kyoto Encyclopedia of Genes and Genomes (KEGG) enrichment analysis of the mRNAs in the network revealed greater enrichment of immune-related pathways, such as the immune system, signal transduction, cell growth and death, and signaling molecules and interactions. We confirmed the selected immune-related DEMs and their DEGs by conducting quantitative RT-PCR (qRT-PCR) analysis. These findings increased our understanding of the interactions of miRNAs and their target genes during infection with REV-SNV, and contributed to the understanding of host-virus interactions.

## Introduction

Avian reticuloendotheliosis virus (REV) is a gamma retrovirus that can cause immunosuppression, dwarf syndrome and acute reticular cell tumors in poultry (Wang et al., 2012; Witter and Glass, 1984). Spleen necrosis virus (SNV) is a type C retrovirus isolated from ducks. It can kill ducklings within one week of inoculation. Phylogenetically, SNVs are grouped among the REV subspecies. The REV strain was first isolated from turkey tumors in the United States in 1958 (Robinson and Twiehaus, 1974). REV is widely distributed in different regions and is reported in countries such as the United States, China, Australia, and Canada (MacDonald et al., 2019; Niewiadomska and Gifford, 2013; Singh et al., 2003). In flocks infected by REV, the vaccinations for Avian influenza, Newcastle disease, Fowl pox and Marek's disease have failed due to immunosuppression (Sun et al., 2009), which has caused significant economic losses in the poultry industry and may threaten human health (Cheng et al., 2007).

MicroRNAs (miRNAs) belong to a class of small noncoding RNAs with a size of 20–25 nucleotides. They play important roles in gene regulation by regulating post-transcriptional silencing of target genes (Lu and Rothenberg, 2018). They are major regulators of many cellular processes, including cell proliferation, differentiation, and apoptosis (Kargutkar et al., 2023). Changes in the expression of specific miRNA genes can lead to inflammation, cancer, and other diseases (Tsuchiya et al., 2006).

Infection by REV damages immune organs, including the spleen, bursa of Fabricius, and thymus. Our previous studies revealed that REV infection induces changes in the spleen transcriptome, including changes in metabolic pathways, immune response processes, and genes involved in tumorigenesis (Gao et al., 2019a; Gao et al., 2019b). The bursa of Fabricius is an important central immune organ of birds; it is the site where the target B cells are reached during virus infection. The REV-T strain can differentially express 88 miRNAs in the bursa of Fabricius (Yu et al., 2017). Therefore, we studied the bursa of Fabricius to assess the effects of REV-SNV infection on different immune organs.

Sequencing the whole transcriptome via next-generation sequencing (NGS) has become efficient, sensitive, and inexpensive over the years (Chang et al., 2011). Therefore, we used specific pathogen-free (SPF) chickens artificially infected with REV-SNV, collected bursa of Fabricius tissues from SPF chickens infected with REV for NGS, and conducted a comprehensive analysis of the overall changes in mRNA and miRNA expression. By screening the miRNA-mRNA interaction network to determine the functional relationship between miRNAs and mRNAs, our study might help elucidate the molecular mechanism by which REV infection leads to immunosuppression and tumor disease.

## Materials and methods

### Ethical statement

The animal tests involved were carried out in accordance with national and institutional guidelines. This study was conducted in strict accordance with the regulations of the Animal Protection and Utilization Committee of Shandong Agricultural University (No. SDAUA-2018-165).

### Animal test design and sample collection

The REV strain SNV (GenBank: DQ003591.1) was provided by Professor Shuhong Sun and Zhizhong Cui of Shandong Agricultural University. The SPF chickens used in this study were purchased from Jinan SAIS Poultry Co. Ltd., China. SPF chickens (n = 40; one day old) were randomly divided into a control group (group C) and a REV-SNV-infected group (group I). The chickens in group I were intraperitoneally injected with 0.2 mL containing 3000 TCID50, and the chickens in group C were intraperitoneally injected with 0.2 mL of DMEM. All chickens were raised in negative pressure filtered air isolators. At 7, 14, and 21 dpi, five chickens in each group were randomly captured and euthanized, and the bursa of Fabricius was collected quickly and aseptically, immediately frozen with liquid nitrogen, and stored at -80 °C the following day.

### Isolation and detection of total RNA

Total RNA was extracted from the bursa of Fabricius samples collected at 7, 14, and 21 dpi after inoculation using the TRIzol kit (Invitrogen, Carlsbad, CA, USA) following the instructions provided with the reagent. Five bursa of Fabricius samples from each group were mixed and divided into three parts for extraction. Total RNA integrity and gDNA contamination were evaluated using 1 % agarose gel electrophoresis, and no degradation or contamination of total RNA was detected. The concentration and purity of total RNA were measured using a spectrophotometer (DS 11, DeNovix, Wilmington, DE, USA). High-purity RNA samples were subsequently used (i.e., OD260/OD280 between 1.9–2.1, OD260/OD230≥2.0, and RNA integrity number ≥7).

Reverse transcription (RT)-polymerase chain reaction (RT-PCR) was performed to test whether SPF chickens were infected with REV-SNV, and the Oligo 7.0 software was used to design long terminal repeat (LTR) fragment primers. The sequences of primers used were as follows: 5′-TCGCTGATATCATTTCTCGGA-3′ LTR forward sequence and 5′-CAGCCAACACCACGAACAAA-3′ LTR reverse sequence. The PCR products were sequenced by TSINGKE Biological Technology (Beijing, China).

### Analysis of sRNA sequencing data

Small RNA (sRNA) libraries were constructed using 2 µg of total RNA from each bursa of Fabricius sample. The sRNA libraries were sequenced by Gene Denovo Biotechnology Co. (Guangzhou, China) using Illumina HiSeq Xten on the Illumina sequencing platform. By screening the raw sequencing data, we removed the reads without inserted fragments and reads with inserted fragment lengths less than 18 nt. Reads with more than one base with a quality value lower than 20 in the data or those that contained N were removed, and reads that contained polyA were removed to obtain sRNA clean tag sequences for the subsequent analysis. Each sample was subsequently analyzed for clean reads with sequence lengths of 18–35 nt, and the selected clean reads were mapped using MiRdeep2 to the gallus reference genome (ftp://ftp.ensembl.org/pub/release-81/fasta/gallus_gallus/dna/). The tag sequences obtained by sequencing were annotated to noncoding RNAs from the GenBank and Rfam databases. Then, the Bowtie (version 1.1.2) software was used to compare the miRNA sequences of this species in the authoritative miRNA database miRbase. Finally, the content of existing miRNAs in the sample and the distribution of bases were obtained. Then, the MiRdeep2 software was used to predict the special secondary structure of the miRNAs and identify new miRNAs.

### Differential miRNA expression analysis prediction of miRNA targets

The miRNAs obtained from each sample were summarized, and the expression level of the TPM (tags per million) of each miRNA is calculated (Zhou et al., 2010). The formula used was TPM = T*10^6^/N (T represents the number of miRNA tags, and N represents the total number of miRNA tags). The expression profiles of all miRNAs in all samples were obtained. The edgeR software was used to analyze differences in miRNA expression between group C and group I. The screening criteria for differentially expressed miRNAs were a more than two-fold change in expression and *p* < 0.05. The target genes of different miRNAs were predicted via Miranda (v3.3a) and TargetScan (version 7.0). The target genes of the differentially expressed miRNAs in each group were significantly enriched according to GO function and KEGG pathway, and the differentially expressed protein genes were identified.

### Transcriptome sequencing and data analysis

The total RNA used for transcriptome sequencing was the same as the total RNA sample used for sRNA sequencing. The total RNA was removed with a Ribo-Zero^TM^ Magnetic Kit (Epicenter, Madison, WI, USA), the mRNA was enriched with Oligo (dT) magnetic beads, the mRNA was cut into short fragments with buffer solution, and random oligonucleotides were used as primers. Double-stranded cDNA was synthesized using a reverse transcriptase system. The double ends of the cDNA were purified and repaired, A base was added, the sequence was connected, and PCR amplification was performed. The library was sequenced by Gene Denovo Biotechnology Co. (Guangzhou, China) using the Illumina NovaSeq 6000 platform.

Reads were filtered using fastp (version 0.18.0) (Chen et al., 2018). Reads containing adapters were removed, reads containing more than 10 % unknown nucleotides (N) were removed, and low-quality reads containing more than 50 % low-quality (Q value≤20) bases were removed. Finally, high-quality clean reads were obtained. The reads were subsequently analyzed against the gallus reference genome using the HISAT2 software. The expression of each gene was calculated based on an FPKM (fragment per kilobase of transcript per million mapped reads) value. Genes with a false discovery rate (FDR) parameter < 0.05 and an absolute fold change ≥2 were considered to be differentially expressed genes.

### Construction of the miRNA-mRNA interaction network

The correlation of expression between miRNAs and mRNAs was evaluated via DEGs and DEMs using the Pearson correlation coefficient (PCC). The negatively correlated miRNA-mRNA pairs were screened by a PCC < –0.7 and *p* < 0.05. A miRNA-mRNA interaction network was constructed, and the mRNAs in the network were analyzed for significant GO functional enrichment and significant KEGG pathway enrichment.

### The expression of differential miRNAs and their target genes was verified by qRT-PCR

We tested the reliability of the high-throughput sequencing results via qRT-PCR detection of common differential miRNAs and their target gene expression levels at the three selected time points. A miRNA 1st Strand cDNA Synthesis Kit (Tail A) (Vazyme, Nanjing, China) was used to synthesize cDNA using the miRNA tailing method with U6 snRNA as the internal reference gene, and the primer sequences of the differential miRNAs are listed in Table 1. For qRT-PCR detection of differential target mRNAs, highly homogeneous cDNA was synthesized for qPCR quantification using a HiScript III 1st Strand cDNA Synthesis Kit (+gDNA wiper) (Vazyme, Nanjing, China). With GAPDH as the internal reference gene, the primer sequences of the target mRNAs are listed in Table 2. The relative levels of expression were normalized to the internal control. Real-time quantitative PCR analysis was performed using a LightCycler®96 (Roche Diagnostics GmbH, Germany) and ChamQ Blue Universal SYBR qPCR Master Mix (Vazyme, Nanjing, China). The reactions were as follows: predenaturation at 95 °C for 30 s, followed by 40 cycles at 95 °C for 10 s and 60 °C for 30 s. Each cDNA sample was divided into three groups, and the qPCR data obtained were analyzed using the 2^–∆∆CT^ method to calculate the relative expression levels of the differentially expressed miRNAs and mRNAs. The primers were designed using the Oligo 7.0 software. All primers used in this study were synthesized by TSINGKE Biological Technology (Beijing, China).

**Table 1:** The miRNAs primers used in this study.

Primers	Sequence (5’–3’)	Size (bp)
gga-miR-215-5p	ATGACCTATGAATTGACAGAC	21
gga-miR-194	TGTAACAGCAACTCCATGTGGA	22
gga-miR-1329-3p	CCTCGTAGCTTGATCACGATAT	22
gga-miR-12224-5p	AACAGTGGAATGCACCTAGCTGA	23
miR-125-x	TCCCGGAGACCCTAACTTGTGT	22
miR-194-x	TGTAACAGCAACTCCATGTGGACA	24
miR-12135-z	TAAGGGTTCGTTTGTAAA	18
miR-215-x	ATGACCTATGAATTGACAGACTT	23
miR-194-z	TGTAACAGCAACTCCATGTGGACC	24
miR-21-z	ATAGCTTATCAGACTGATGTTGACTTT	27
novel-m0035-5p	TCCCTGAGACCCTAACTTGTGATGT	25
novel-m0124-3p	AACAGGCACGACCGAGGCTGAAA	23
U6	TGGAACGCTTCACGAATTTGCG	22

**Table 2:** The mRNAs primers used in this study.

Primers	Sequence (5’–3’)	Size (bp)
TLR3	Forward: CAAGAGCCTGAGAACACTAGAGAT	24
	Reverse: GCCAACTAGCAAACCAAGCAAT	22
CCL19	Forward: GAACAGCAGCAGAACGCAGCAA	22
	Reverse: ACCAGGAGACTAAGGCAGAGAACG	24
CCL1	Forward: GTGCTGCTTCAACTTCCATACCA	23
	Reverse: ATCTCCTTGCCATTCTTCACCTTG	24
CCL4	Forward: GGCAGACTACTACGAGACCAACA	23
	Reverse: TGATGAACACAACACCAGCATGAG	24
STAT1	Forward: CCTCTGGAACGATGGCTGTATC	22
	Reverse: TCCTGGTCTCTGGTCCTTCAATA	23
TRIM25	Forward: TAACCACCACCCTCAGCGTTTC	22
	Reverse: CAGATGCCAATGCCACAGAAGTT	23
C1R	Forward: TTATGGCTTCTACACCAAGATCCTC	25
	Reverse: GGAACACTTAGAGCGTATCCTCC	23
C1S	Forward: CCAATTATCCTCAGGCATACCCAA	24
	Reverse: CTTCACAGAGTCATATTCGCAGT	23
IFIH1	Forward: CTTCCTCTACATGATCTCCTGCTTC	25
	Reverse: CCGCACCTTGTCCTTCTCCT	20
IRF1	Forward: CGTCCATCACCTTAGACCTCTCGT	24
	Reverse: GATTTCCATCGGCATCCTCCAGTC	24
IFNG	Forward: AGCTCCCGATGAACGACTTG	20
	Reverse: TCCTCTGAGACTGGCTCCTT	20
Vpreb2	Forward: TCTCTGGGTATCGGCAGACC	20
	Reverse: AGGTAAATTTCCAGTTGGATGGTG	24
YF5	Forward: CGCTACTTCCTGACCGGGAT	20
	Reverse: CCACGTACCCGACGGC	16
CDKN1A	Forward: GAGCGGTGGAACTTCGACTT	20
	Reverse: CGGGCAAGTAGACCTTAGGC	20
CSF2RB	Forward: AAGGTGGCATTCATCCCAGG	20
	Reverse: GTCCGCACAGACACTTGGTA	20
GADPH	Forward: GCCATCACAGCCACACAGAAGA	22
	Reverse: GGCAGGTCAGGTCAACAACAGA	22

### Statistical analysis

All data were expressed as the mean ± standard deviation. Correlation analysis was performed between the NGS results and the qRT-PCR results using the GraphPad Prism 8.0 software.

## Results

### Verification of REV-SNV infection in SPF chickens

**Fig. 1:**
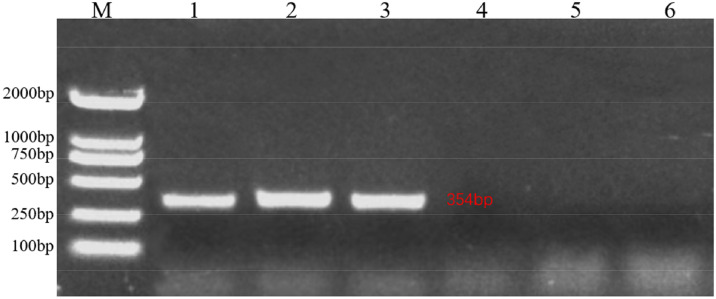
Detection of Reticuloendotheliosis virus-spleen necrosis virus (REV-SNV) RNA from the bursa of Fabricius of infected chickens. The DNA marker (M) used DL2000 (Takara, Dalian China). Lanes 1–3 are the PCR products of the IFN group at 7, 14, and 21 dpi, respectively. Lanes 4–6 are the PCR products of the CON group at 7, 14, and 21 dpi, respectively.

We performed RT-PCR analysis of the RNA extracted from the bursa of Fabricius samples collected from infected chickens and control group SPF chickens. For all samples in the infected group, PCR amplification bands corresponding to the 354 bp region of the TLR region were detected by conducting agarose gel electrophoresis (Fig. 1). After sequencing, we found that the PCR product sequences were identical to those of the REV-SNV (GenBank: DQ003591.1), which had the same LTR region; this finding confirmed that REV-SNV infection occurred in the infected group.

### mRNA-seq analysis of 18 bursa of Fabricius samples

We performed mRNA sequencing of 18 bursa of Fabricius samples, including six 7 dpi samples (con1-1, con1-2, con1-3, inf1-1, inf1-2, and inf1-3), six 14 dpi samples (con2-1, con2-2, con2-3, inf2-1, inf2-2, and inf2-3), and six 21 dpi samples (con3-1, con3-2, con3-3, inf3-1, inf3-2, and inf3-3). The sequencing data for each library were obtained (Table 3). According to the Q30 value of each library, the sequencing quality was satisfactory.

**Table 3:** Summary of the mRNA sequencing data.

Sample	Raw Reads	Clean Reads	Q30 (%)	Mapped Reads
con1-1	39,627,354	39,455,620	91.61	36,578,916
con1-2	41,827,660	41,644,666	91.17	38,622,712
con1-3	39,583,016	39,418,100	91.48	36,562,124
con2-1	36,500,850	36,333,766	91.67	33,251,063
con2-2	40,097,828	39,916,340	91.92	36,641,599
con2-3	37,783,924	37,601,556	90.72	34,610,802
con3-1	41,622,852	41,430,134	91.77	37,802,318
con3-2	40,301,080	40,096,484	92.03	36,178,414
con3-3	36,973,054	36,792,912	91.68	33,190,554
inf1-1	36,944,854	36,764,404	91.43	33,743,420
inf1-2	40,045,672	39,875,062	91.59	36,568,349
inf1-3	39,562,718	39,393,168	91.92	36,191,496
inf2-1	39,395,674	39,200,534	90.59	35,439,994
inf2-2	40,186,518	40,021,048	91.95	36,439,797
inf2-3	46,801,530	46,629,754	94.30	42,382,317
inf3-1	40,118,624	39,918,508	90.96	36,241,902
inf3-2	40,162,770	40,004,820	92.47	36,586,563
inf3-3	39,918,224	39,719,964	91.40	35,956,491

### DEG analysis

**Fig. 2:**
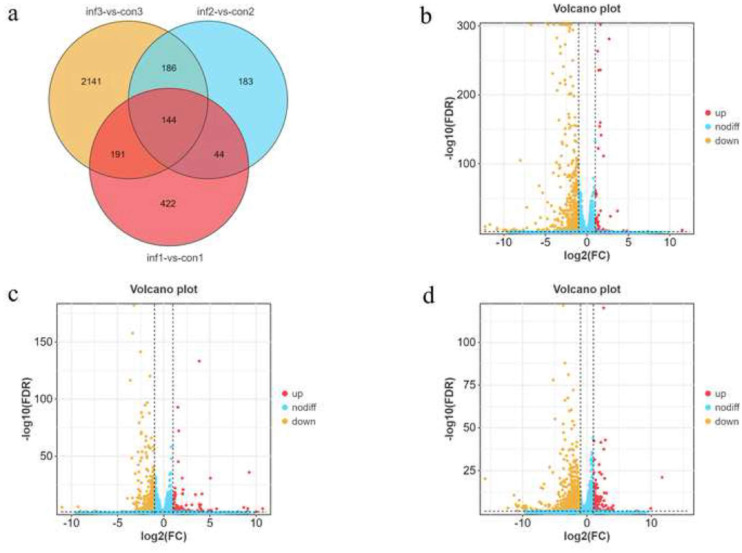
Venn diagrams. (a) Number and overlap of differential mRNAs during REV infection of the bursa of Fabricius at 7, 14, and 21 dpi. Venn diagram analysis revealed 144 mRNAs at all three tested time points. Volcano plot of differential mRNA expression at 7 (b), 14 (c), and 21 dpi (d). The x-axis represents log2 (FC), and the y-axis represents -log10 (P-Value). The red points represent the upregulated mRNAs, whereas the yellow points represent the downregulated mRNAs.

The genes identified by an FDR < 0.05 and an absolute fold change ≥2 were considered to be DEGs. We identified 3311 DEGs in the CON and INF groups at 7, 14, and 21 dpi, and 144 DEGs at the intersection of the three time points (Fig. 2a). In the 7 dpi samples, 801 genes were identified, including 76 genes whose expression was upregulated and 725 genes whose expression was downregulated (Fig. 2b). In the samples collected at 14 dpi, 557 genes were identified, including 120 upregulated genes and 437 downregulated genes (Fig. 2c). Additionally, 2662 genes were identified in the 21 dpi samples, including 199 genes whose expression was upregulated and 73 downregulated (Fig. 2d). There were considerably more downregulated genes than upregulated genes at the three time points. Overall, the REV-SNV infection significantly affected the overall gene expression profile.

### miRNA-seq analysis of 18 bursa of Fabricius samples

**Fig. 3:**
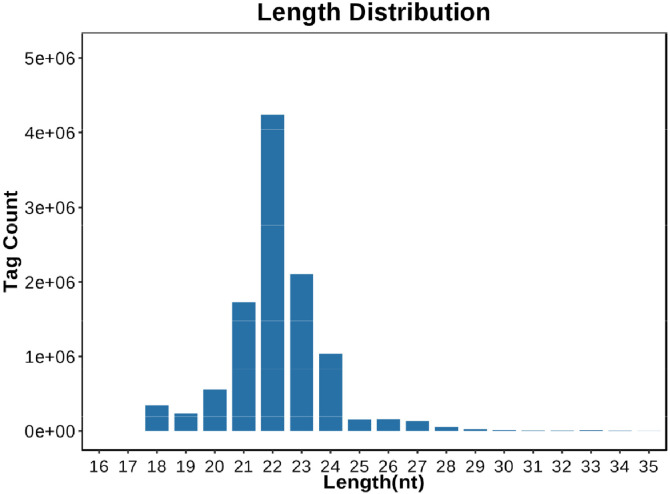
Length distribution results for the miRNA sequences. Only the micon1-1 example results are shown. The x-axis represents the tag length (nt), and the y-axis represents the tag count.

We performed miRNA sequencing of 18 bursa of Fabricius samples, including six 7 dpi samples (micon1-1, micon1-2, micon1-3, miinf1-1, miinf1-2, and miinf1-3), six 14 dpi samples (micon2-1, micon2-2, micon2-3, miinf2-1, miinf2-2, and miinf2-3), and six 21 dpi samples (micon3-1, micon3-2, micon3-3, miinf3-1, miinf3-2, and miinf3-3). Sequencing data for each library were obtained (Table 4). According to the high-quality values of each library, the sequencing quality was satisfactory. Most tags were 21–24 nt long with only one peak and were most abundant at 22 nt (Fig. 3), which was similar to our previous results (Gao et al., 2019a). These results indicated that miRNAs were continuously enriched from 18 libraries.

**Table 4:** Summary of the small RNA sequencing data after filtering and mapping.

Sample	Raw reads	Clean reads	High quality (%)	Select Reads	Select match reads	Match rate (%)
micon1-1	12,526,399	12,423,667	98.18	10,817,135	8,680,793	80.25
micon1-2	10,569,801	8,783,634	83.10	7,776,622	6,249,663	80.36
micon1-3	10,264,296	8,521,326	83.02	7,555,692	6,062,764	80.24
micon2-1	12,510,979	10,394,623	83.08	8,901,046	7,114,241	79.93
micon2-2	15,971,434	15,765,473	98.71	12,709,263	9,931,633	78.14
micon2-3	10,872,348	10,775,949	99.11	8,732,636	6,899,962	79.01
micon3-1	11,234,583	11,137,531	99.14	8,691,313	6,557,437	75.45
micon3-2	14,608,276	14,515,872	99.37	11,269,495	8,665,529	76.89
micon3-3	10,553,849	10,443,309	98.95	8,069,984	6,132,833	76.00
miinf1-1	16,494,810	16,302,765	98.84	13,708,362	10,743,247	78.37
miinf1-2	9,660,583	8,031,437	83.14	7,024,494	5,465,584	77.81
miinf1-3	10,354,732	10,255,681	99.04	8,714,976	6,851,878	78.62
miinf2-1	10,691,056	10,601,093	99.16	9,663,632	7,528,952	77.91
miinf2-2	10,285,633	8,559,321	83.22	7,925,254	6,284,548	79.30
miinf2-3	9,881,826	8,208,124	83.06	7,447,739	5,806,634	77.97
miinf3-1	9,439,328	9,354,114	99.10	7,827,974	5,895,044	75.31
miinf3-2	16,767,435	16,570,405	98.82	13,391,698	10,148,619	75.78
miinf3-3	12,322,477	12,210,476	99.09	10,401,489	7,892,616	75.88

### DEM analysis

**Fig. 4:**
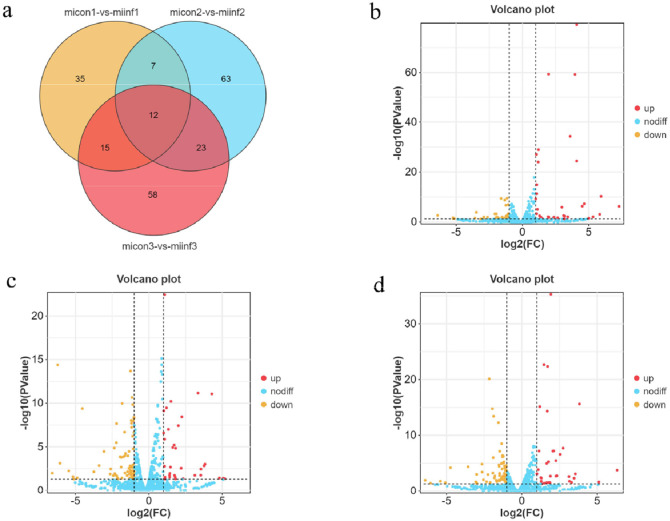
Venn diagrams. (a) Number and overlap of differential miRNAs during REV infection of the bursa of Fabricius at 7, 14, and 21 dpi. Venn diagram analysis revealed 12 miRNAs at all three tested time points. Volcano plot of differential miRNA expression at 7 (b), 14 (c), and 21 dpi (d). The x-axis represents log2 (FC), and the y-axis represents -log10 (P-Value). The red points represent the upregulated miRNAs and yellow points represent the downregulated miRNAs.

Using the edgeR software, normalization and statistical analysis were performed based on miRNA sequences. We identified 213 differentially expressed miRNAs (DEMs) in the CON and INF groups at 7, 14, and 21 dpi and 12 DEMs at the intersection of the three time points (Fig. 4a). Among the samples collected at 7 dpi, 69 DEMs were identified, including 35 upregulated and 34 downregulated miRNAs (Fig. 4b). Among the samples collected at 14 dpi, 105 were identified, including 34 upregulated and 71 downregulated miRNAs (Fig. 4c). Additionally, 108 miRNAs were identified in the 21 dpi samples, including 35 upregulated and 73 downregulated miRNAs (Fig. 4d).

### Functional enrichment analysis of differential miRNA expression

**Fig. 5:**
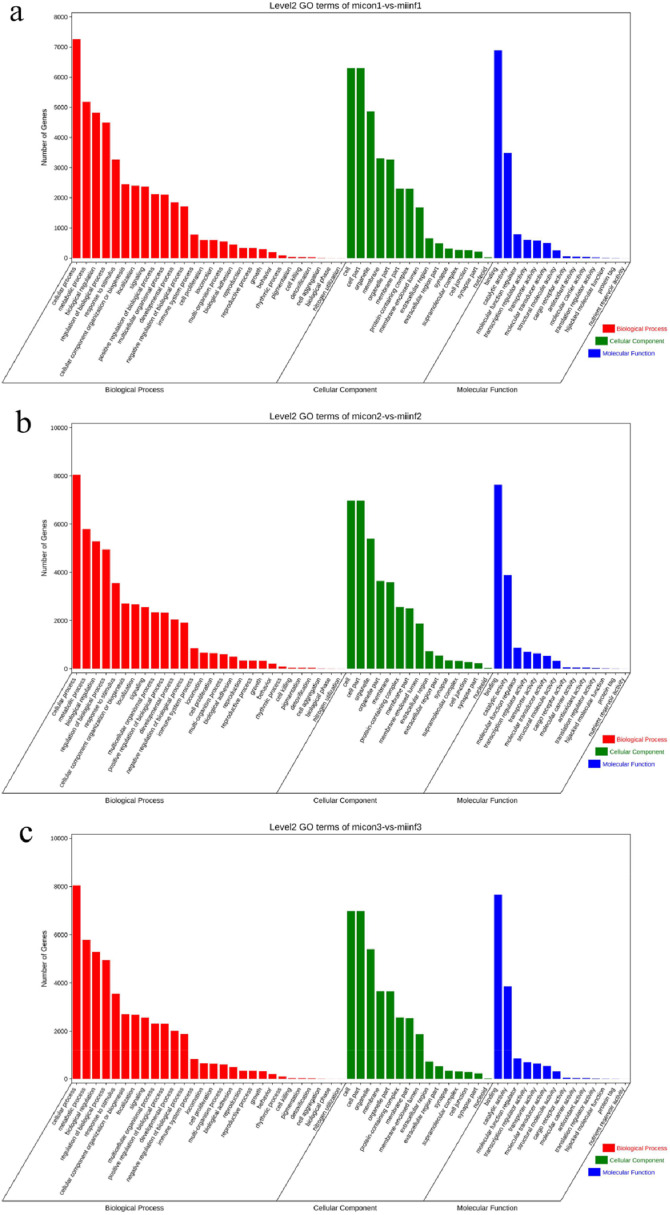
Functional enrichment analysis of candidate target genes at 7 (a), 14 (b) and 21 dpi (c). The x-axis represents the second-order GO term, and the y-axis represents the number of miRNA target genes in this term. Different colors indicate different types of GO terms.

To determine the biological functions of different miRNAs in host chickens infected with REV-SNV, two methods, Miranda (v3.3a) and TargetScan (version 7.0), were used to predict candidate target genes. GO functional significance enrichment analysis was performed to identify the major biological functions of the target genes of the differentially expressed miRNAs. The results of the GO analysis (Fig. 5a-c) revealed that the genes associated with the GO biological process (GO-BP) category were enriched mainly in cellular terms “cellular process”, “metabolic process”, “regulation of biological process”, “response to stimulus”, “cellular component organization or biogenesis”, and “immune system process”. The GO cell component (GO-CC) category mainly included the terms “cell part”, “organelle part”, “membrane part”, “protein-containing complex”, “membrane-enclosed lumen”, and “extracellular region part”. The GO molecular function (GO-MF) category included the terms “binding”, “catalytic activity”, “molecular function”, “regulator, transcription regulator activity”, “transporter activity”, and “molecular transducer activity”.

**Fig. 6:**
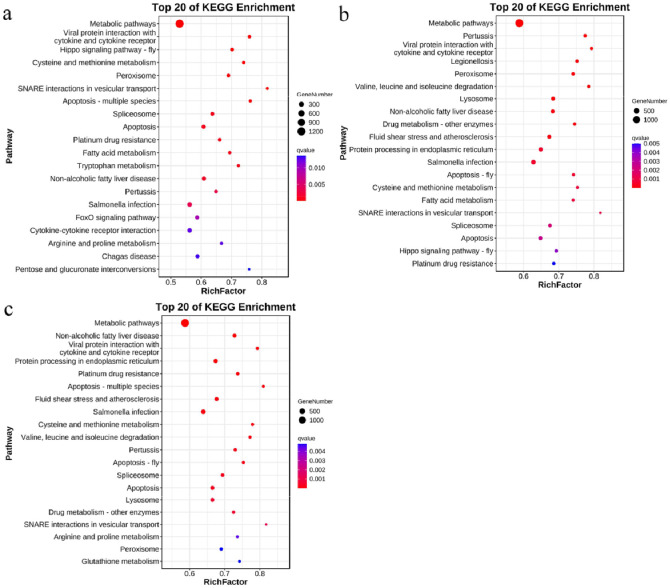
Kyoto Encyclopedia of Genes and Genomes (KEGG) pathway analysis of candidate target genes at 7 (a), 14 (b), and 21 dpi (c). The first 20 pathways with the smallest Q values were mapped. The y-axis represents the pathway, and the x-axis represents the enrichment factor. The size represents the number of miRNA target genes; the redder the color, the smaller the Q value.

The most significant biochemical metabolic pathways and signal transduction pathways of the miRNA candidate genes at 7, 14, and 21 dpi were determined by conducting the Kyoto Encyclopedia of Genes and Genomes (KEGG) enrichment analysis. The results of the KEGG analysis (Fig. 6a-c) revealed that many of the genes enriched by the KEGG pathway were associated with Metabolic pathways, Viral protein interaction with cytokine and cytokine receptors, Lysosome, SNARE interactions in vesicular transport, Hippo signaling pathway-fly, Apoptosis and Protein processing in endoplasmic reticulum were significantly correlated. These results suggested that DEMs may play important roles in virus-host interactions when chickens are infected with REV-SNV.

### miRNA-mRNA integrated analysis

The negative correlations and interaction networks of differential miRNAs and differential target mRNAs were comprehensively analyzed to provide valuable insights into the role of DEMs in the process of REV infection. Based on the 213 DEMs and 3311 DEGs screened in the previous steps, 1376 miRNA-mRNA interaction pairs were obtained, involving 84 DEMs and 610 DEGs, according to the criteria of negative correlation of expression levels and targeted relationships. The results revealed that most miRNAs have multiple target genes.

**Fig. 7:**
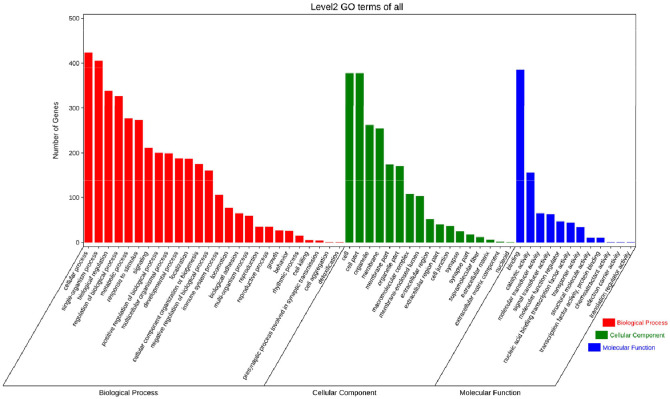
Functional enrichment analysis of target genes. The x-axis is the second-order GO term, and the y-axis is the number of miRNA target genes in this term. Different colors indicate different types of GO terms.

The functional enrichment analysis of miRNA-mRNA interactions with target mRNAs provided insights into their functional role during chicken infection with REV-SNV. The results of the GO analysis (Fig. 7) revealed that the GO biological process (GO-BP) category was enriched in mainly included the terms “cellular process”, “metabolic process”, “regulation of biological process”, “response to stimulus”, “cellular component organization or biogenesis” and “immune system process”. The GO cell component (GO-CC) category mainly included the terms “cell part”, “organelle”, “membrane part”, “macromolecular complex”, “membrane-enclosed lumen”, and “extracellular region part”. The GO molecular function (GO-MF) category included “binding”, “catalytic activity”, “catalytic activity”, “molecular function regulator”, “nucleic acid binding transcription factor activity”, “transporter activity”, and “transcription factor activity, protein binding”.

**Fig. 8:**
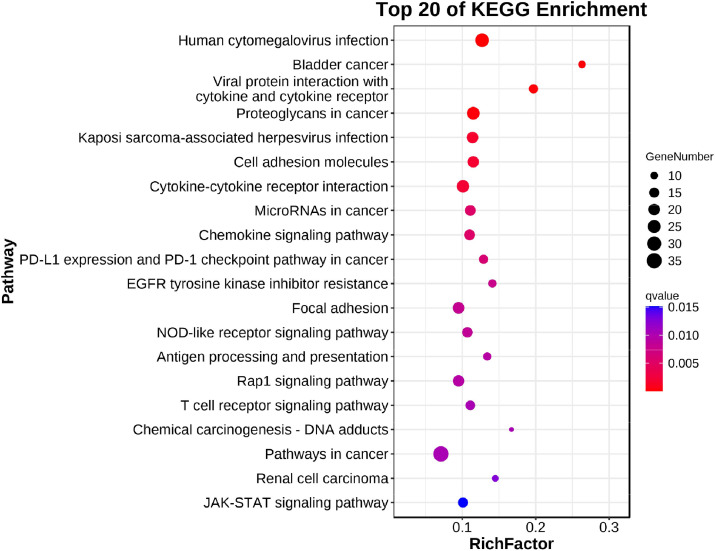
KEGG pathway analysis of target genes. The first 20 pathways with the smallest Q values were mapped. The y-axis represents the pathway, and the x-axis represents the enrichment factor (the number of miRNA target genes in this pathway divided by all the numbers in this pathway). The size represents the number of miRNA target genes; the redder the color, the smaller the Q value.

**Fig. 9:**
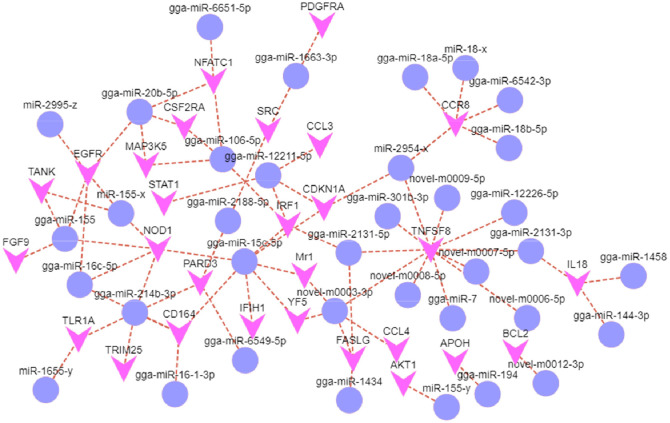
Network diagram of miRNAs and negatively correlated target genes involved in immunity. The v shapes represent genes, and the circles represent miRNAs that target those genes.

The results of the KEGG analysis (Fig. 8) revealed that a large number of genes enriched by KEGG were associated with Viral protein interaction with cytokine and cytokine receptor, Proteoglycans in cancer, Cell adhesion molecules, Cytokine-cytokine receptor interaction, Chemokine signaling pathway, PD-L1 expression and PD-1 checkpoint pathway in cancer, NOD-like receptor signaling pathway, Rap1 signaling pathway, T cell receptor signaling pathway, Pathways in cancer, and JAK-STAT signaling pathway were significantly correlated. Using the Cytoscape network construction software, known miRNAs and mRNAs related to signal transduction, signaling molecules and interaction, transport and catabolic, cell growth and death, immune system and other aspects with negative correlations were visualized (Fig. 9).

### qRT-PCR validated significant DEMs and DEGs

**Fig. 10:**
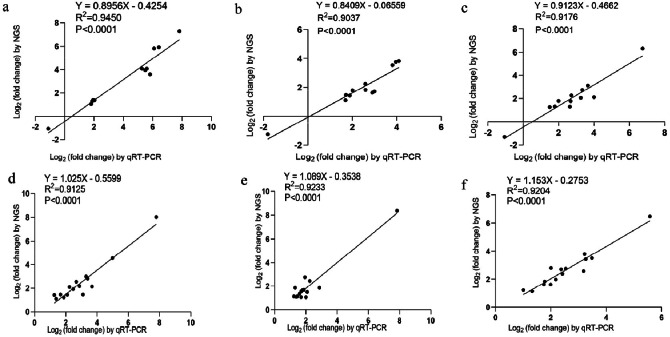
The DEMs and DEGs were confirmed by conducting by quantitative RT-PCR (qRT-PCR). (a-c) Correlation analyses of DEMs expression at 7, 14, and 21 dpi, respectively. (d-f) The level of expression of the DEGs at 7, 14, and 21 dpi, respectively. The x-axis represents the log2 (fold change) by qRT-PCR, and the y-axis represents the log2 (fold change) by NGS.

Quantitative RT-PCR was performed to validate the differentially expressed miRNAs and mRNAs in the NGS data. The 12 DEMs at the intersection of 7, 14, and 21 dpi were selected (gga-miR-215-5p, gga-miR-194, gga-miR-1329-3p, gga-miR-1329-3p, miR-125-x, miR-125-x, miR-12135-z, miR-215-x, miR-194-z, and miR-21-z). The gene expression changes in the 12 DEMs were comparable among the three time points, with correlation coefficients of R^2^ = 0.9450 (*p* < 0.0001), 0.9037 (*p* < 0.0001), and 0.9176 (*p* < 0.0001) (Fig. 10a-c).

The 15 DEGs at the intersection of 7, 14, and 21 dpi were selected (TLR3, CCL19, CCL1, CCL4, STAT1, TRIM25, C1R, C1S, IFIH1, IRF1, IFNG, Vpreb2, YF5, CDKN1A, CSF2RB). The gene expression changes of the 15 DEGs were comparable among the three time points, with correlation coefficients of R^2^ = 0.9125 (*p* < 0.0001), 0.9233 (*p* < 0.0001), and 0.9204 (*p* < 0.0001) (Fig. 10d-f). The results of qRT-PCR assays were consistent with those of high-throughput sequencing, which indicated that the sequencing data used in this study were reliable.

## Discussion

High-throughput sequencing technology can be used for diagnosing diseases in plants and animals and for detailed studies of host-pathogen interactions at the genomic and transcriptome levels (Head et al., 2014; Van Borm et al., 2015). Through integrated analysis, the expression profiles of miRNAs and the genome in plant and animal tissues can be revealed to investigate the relationships between miRNAs and mRNAs, which has important reference significance for the disease and biology of animals and plants (Jia et al., 2024; Ortega-Acosta et al., 2024; Wu et al., 2020). REV-infected chickens attack mainly B-cell-mediated and T-cell-mediated immune organs and severely affect the immune functions of the spleen, bursa of Fabricius, and thymus (Xu et al., 2020). We previously studied changes in the miRNA and mRNA expression profiles of the spleen of SPF chickens infected with REV-SNV (Gao et al., 2019a; Gao et al., 2019b; Jiang et al., 2021). In chickens, the spleen is a peripheral immune organ, but the bursa of Fabricius is an important central immune organ and the main site of B lymphocyte development and differentiation (Zhang et al., 2023). To assess the effects of REV-SNV infection on different immune organs, in this study, we analyzed the expression profiles of miRNAs and mRNAs in the bursa of Fabricius of SPF chickens at 7, 14, and 21 dpi post REV-SNV infection to identify miRNAs and target genes that respond to REV-SNV. We constructed miRNA-mRNA interaction networks based on the screened DEMs and DEGs. The differential expression of 213 miRNAs and 3311 mRNAs in the bursa of Fabricius in REV-SNV-infected SPF chickens was identified, and 1376 negatively correlated miRNA-mRNA pairs were constructed, of which 82 pairs were identified at 7 dpi, 203 pairs were identified at 14 dpi, and 873 pairs were identified at 21 dpi. These results suggested that as the age of chickens increases, their immune system continues to develop and improve, and infected chickens exhibit increasingly strong immune protection responses against virus invasion. Some single miRNAs can target multiple genes. For example, gga-miR-214b-3p can simultaneously target PARD3 (par-3 family cell polarity regulator), TLR1A (toll-like receptor 1 family member), TRIM25 (tripartite motif containing 25), NOD1 (nucleotide binding oligomerization domain containing 1), and CD164 (CD164 molecule). A single gene can be targeted by multiple miRNAs, such as TNFSF8 (tumor necrosis factor superfamily member 8), which can be simultaneously targeted by gga-miR-2131-5p, gga-miR-301b-3p, and gga-miR-2131-3p.

By regulating the expression of target genes, miRNAs participate in various regulatory pathways, including those involved in viral replication, cell proliferation, differentiation, apoptosis, the antiviral immune response, and tumorigenesis (Fani et al., 2018; Gottwein and Cullen, 2008; Trobaugh and Klimstra, 2017). Many researchers have studied miRNAs at a greater depth and provided new insights into the study of host-virus interactions (Chen et al., 2020; Zhuo et al., 2013). Hongmei Li performed miRNA microarray analysis of the livers of 10-week-old chickens infected with avian leukosis virus (ALV) and identified 12 miRNAs with significant expression (Li et al., 2012). Mohammad Heidari performed miRNA sequencing on 21-day MD-resistant and MD-susceptible chickens in the bursa of Fabricius tissues infected with Marek's disease virus (MDV); they identified 54 new miRNAs and 10 known miRNAs. Several immune-related pathways, including the Toll-like receptor signaling pathway and other pathways, were identified through enrichment analysis (Heidari et al., 2020). To highlight the miRNAs associated with REV-SNV interactions with SPF chickens, we enriched mRNAs in the miRNA-mRNA negatively correlated networks to study their molecular mechanisms and biological functions. The analysis of the KEGG pathways of 610 mRNAs in the negatively correlated network of miRNA-mRNA revealed that the genes associated with this network were enriched mainly with Viral protein interaction with cytokine and cytokine receptor Proteoglycans in cancer and Cell adhesion molecules, Cytokine-cytokine receptor interaction, Chemokine signaling pathway, PD-L1 expression and PD-1 checkpoint pathway in cancer, Rap1 signaling pathway, T cell receptor signaling pathway, Pathways in cancer, JAK-STAT signaling pathway. Previous studies have reported that these pathways are involved in host antiviral processes (Goenka et al., 2023; Tzeng et al., 2021; Zheng, 2021). Overall, miRNAs influence the regulation of the host immune response, which may be related to the replication of REV-SNV during early infection in SPF chickens.

In the interaction network of miRNA-mRNA, TLR1A is negatively correlated with gga-miR-214b-3p and miR-1655-y. It is involved in the recognition of virus invasion by Toll-like receptor (TLR) and initiates the innate immune response through a series of signal transduction pathways (Bovenhuis et al., 2022; Kawai and Akira, 2007). CCR8 is negatively correlated with gga-miR-18b-5p, gga-miR-18a-5p, gga-miR-6542-3p, miR-2954-x, and miR-18-x and is involved in viral protein interaction with cytokine and cytokine receptor as chemokine receptors that are highly expressed on regulatory T cells (Tregs) (Suzuki et al., 2022). CCR8 often acts synergistically with other chemokine systems, as well as immune regulatory cytokines and growth and differentiation factors, to inhibit/promote tumors (Moser, 2022). NOD1 is negatively correlated with gga-miR-16c-5p, gga-miR-155, gga-miR-15c-5p, gga-miR-214b-3p, and miR-155-x, which participate in the NOD-like receptor signaling pathway. This pathway plays an important role in host innate immunity, regulating cell proliferation and apoptosis, autophagy, and the inflammatory response (Kuss-Duerkop and Keestra-Gounder, 2020; Trindade and Chen, 2020). CDKN1A is negatively correlated with gga-miR-15c-5p, gga-miR-12211-5p, and miR-2954-x. CDKN1A (p21 gene) is a member of the cell cycle protein-dependent kinase inhibitor family, which is involved in cell cycle regulation, cell migration, differentiation, and apoptosis. It is also associated with the occurrence and metastasis of tumors (Kreis et al., 2019; Xiao et al., 2020). EGFR is negatively correlated with gga-miR-16c-5p, gga-miR-155, gga-miR-20b-5p, miR-155-x, and miR-2995-x and is a key regulator of cell proliferation, growth, differentiation, and cancer development (Sabbah et al., 2020; Talukdar et al., 2020). EGFR plays a key role in regulating signaling processes during the cell cycle and is an essential component, considering that it can promote mitotic and carcinogenic effects (Lui and Grandis, 2002). LCK is negatively correlated with gga-miR-2188-5p, gga-miR-15c-5p, gga-miR-1456-5p, gga-miR-214-3p, and miR-347-z and plays a key role in T lymphocyte differentiation and activation. It can regulate the initiation of the T-cell receptor signaling pathway (TCR), T-cell development, and T-cell homeostasis (Bommhardt et al., 2019; Isakov and Biesinger, 2000). IRF1 is negatively correlated with gga-miR-106-5p, gga-miR-2131-5p, gga-miR-15c-5p, and gga-miR-12211-5p and plays an important role in protecting the host from pathogen invasion and maintaining homeostasis in the host, acting as the main regulator of innate immunity. Several studies have shown that IRF1 is associated with tumor suppression, the development of natural killer (NK) cells and T-cells, and B-cell biology (Feng et al., 2021; Mboko et al., 2015; Yamada et al., 1991).

To summarize, these results suggested that these miRNAs can regulate the function of target genes and promote or inhibit the replication of REV-SNV by targeting important regulators of immune-related signaling pathways.

## Author contributions

Conceived and designed the experiments and reviewed the article: YXD YT JDH YBZ. Performed the experiments and wrote the paper: YBZ. Analyzed the data: YBZ and QZ. QZ, MW, BRW, SSZ, and XHW helped YBZ with animal husbandry and sample collection.

## Declaration of competing interest

The authors declare that the research was conducted without any commercial or financial relationships that could be construed as potential conflicts of interest.
